# Linking Macroscopic with Microscopic Neuroanatomy Using Synthetic Neuronal Populations

**DOI:** 10.1371/journal.pcbi.1003921

**Published:** 2014-10-23

**Authors:** Calvin J. Schneider, Hermann Cuntz, Ivan Soltesz

**Affiliations:** 1 Department of Anatomy and Neurobiology, University of California Irvine, Irvine, California, United States of America; 2 Ernst Strüngmann Institute (ESI) for Neuroscience in Cooperation with Max Planck Society, Frankfurt/Main, Germany; 3 Institute of Clinical Neuroanatomy, Goethe University, Frankfurt/Main, Germany; 4 Frankfurt Institute for Advanced Studies, Frankfurt/Main, Germany; Hamburg University, Germany

## Abstract

Dendritic morphology has been shown to have a dramatic impact on neuronal function. However, population features such as the inherent variability in dendritic morphology between cells belonging to the same neuronal type are often overlooked when studying computation in neural networks. While detailed models for morphology and electrophysiology exist for many types of single neurons, the role of detailed single cell morphology in the population has not been studied quantitatively or computationally. Here we use the structural context of the neural tissue in which dendritic trees exist to drive their generation *in silico*. We synthesize the entire population of dentate gyrus granule cells, the most numerous cell type in the hippocampus, by growing their dendritic trees within their characteristic dendritic fields bounded by the realistic structural context of (1) the granule cell layer that contains all somata and (2) the molecular layer that contains the dendritic forest. This process enables branching statistics to be linked to larger scale neuroanatomical features. We find large differences in dendritic total length and individual path length measures as a function of location in the dentate gyrus and of somatic depth in the granule cell layer. We also predict the number of unique granule cell dendrites invading a given volume in the molecular layer. This work enables the complete population-level study of morphological properties and provides a framework to develop complex and realistic neural network models.

## Author Summary

Computational models of neurons and neural networks provide a valuable avenue to test our understanding of brain regions and to make predictions to guide future experimentation. Each neuron has a unique dendritic tree, features of which can vary depending on the location of the neuron within the particular brain region. In this study, we generated a complete population of dendritic trees for the most numerous type of neuron in the hippocampus, the dentate gyrus granule cell, using a realistic three-dimensional structural context to drive the generation process. Morphological properties can now be studied at the level of complete neuronal populations, and this work provides a foundation to build upon in the construction of large-scale, data-driven neuroanatomical and network models.

## Introduction

Growing evidence for the importance of dendritic structure on neuronal function has inspired the construction of morphologically realistic computational models of single neurons. Dendritic morphology has been shown to have a significant impact on neuronal firing properties, both between neurons of different classes [1] and within the same class [2], [3], as well as on signal integration and propagation [4]–[6]. The intra-class morphological variability could have a significant impact on the integration of individual neurons into the circuit and their resulting role in network computation. Correspondingly, this has led to the development of detailed three-dimensional morphological reconstructions of single cells [7] and functional models incorporating this level of detail [8], [9]. Not only does the incorporation of realistic morphology enable more accurate reproduction of measured electrophysiology, it also allows for a more detailed representation of network connectivity. These together enable a better understanding of the underlying computation in the network. Advances in computational power as well as in parallel computing, such as the development of parallel versions of neurophysiological simulation environments [10]–[14], have made the simulation of large networks with detailed neuron models accessible. Currently, however, the majority of functional electrophysiological network models utilize uniform single models or very small subsets of models to describe neurons of a given class, overlooking the inherent biological diversity. In addition, the connectivity is usually oversimplified in almost all functional neural networks, whether by the use of probabilistic methods rather than explicit connectivity or by making connections using only a subset of the neurons in the network population, while in most applications it should in fact reflect the full morphological architecture of dendrites and axons. The generation of full-scale, population-level morphological models is, therefore, an important and timely goal. Since experimental reconstructions are to date available only in small sample sizes, techniques to generate population-level morphological models will require the amplification of these data sets to fully realistic and diverse populations [15].

Aside from quantifications based on reconstructions of single cells, existing neuroanatomical data encompasses a large number of measures at multiple levels, such as density estimates for synaptic zones using electron microscopy or cell counts and population analysis using molecular techniques as well as entirely macroscopic features of neural tissue [16]. The optimal arrangement of elements of neural circuits in the brain has been extensively studied [17]–[21], and a recent trend has been to put neuroanatomical single cell reconstructions in the macroscopic context in which they originally existed [22]–[24]. In particular, recent work in *Drosophila* has focused on generating a standardized structural model [25] and taken steps toward generating a complete network connectivity map by placing all reconstructed neurons into a standard brain [26], which is possible given the smaller population of neurons in invertebrate model organisms. Conventional light microscopy does not have the resolution to reconstruct circuits in densely labeled neuropil [27], and as a result, modern techniques such as large-scale serial block-face scanning electron microscopy have started to provide reconstruction methods for which both the microscopic details of all cells and the macroscopic circuit-level features are present in the same biological tissue samples [28]–[30]. These data, however, are rather large and complex, and it will be important to develop novel approaches to facilitate the study of such neuroanatomical connectomes [31]. The development of large-scale morphological models with macroscopic constraints will enable the analysis of these large data sets and the study of connectomes before full anatomical reconstructions are available.

Current methodologies for the generation of morphological models primarily employ reconstructions and their branching characteristics independent of their originating context. Several studies have relied solely on the reconstructions themselves, involving pure duplication [32], making small variations in the lengths and angles of tree branches [33], or resizing to fit within a spatial context [24]. Other methods have focused on the branching properties of the reconstructions and have used a wide variety of algorithms, including the simulation of growth cones with NETMORPH [34], modeling self-referential forces [35], or mapping one-dimensional structures to 3D trees [36]. Several of these tools, such as L-Neuron [37], EvOL-Neuron [38], and NeuGen [39], [40], create variable dendritic trees by stochastically sampling branching parameters from extracted statistical distributions. While the stochastic sampling methodology is able to generate realistic synthetic trees, it was too inefficient in our previous work to generate a complete and distributed population [15], even without the constraint of fitting within a three-dimensional context. The current study reverses the direction of previous methods by starting with the macroscopic neuroanatomy and enables complete population-level construction and analysis.

Here we report a method that allows us to match generated single cell morphologies to measured data as a function of macroscopic features. We do this by devising a computational model that generates morphologies of all single neurons in a population while considering the broader neuroanatomical context in which they grow. First we model the volume of the rat dentate gyrus based on a recent detailed reconstruction of the entire structure [24]. We then generate single cell morphologies of all granule cells (GCs) as described previously [41] constrained within this volume. The resulting population data matches the known variability in GC morphology as well as some known key dependences of GC features on location within the dentate gyrus. We then use our model to develop measures and predictions for dendritic features at the population level. This work provides a valuable framework for the study of complete populations of neuronal morphologies and represents a major step in the development of large-scale neural network models.

## Results

### Model dentate gyrus boundaries

**Figure 1:**
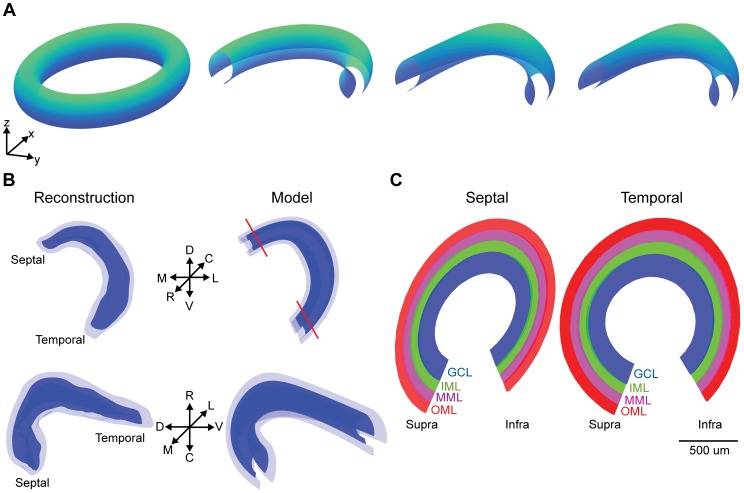
Parametric volume representing the dentate gyrus structure. (**A**) The outer boundary for the granule cell layer was created by cutting a piece out of an elliptical torus (left) into a partial structure (middle left), then adding a deflection in the z direction (middle right), and finally creating unequal septal and temporal ends (right). See Methods for details. (**B**) Comparison between the experimental reconstruction and model structure. The rostrocaudal, dorsoventral, and mediolateral axes are shown. Red lines depict slice angles for (C). (**C**) 100 µm transverse slices of septal and temporal regions at the level of the red lines in (B). Subdivisions into the different layers is indicated by colors: GCL – granule cell layer (blue), IML – inner molecular layer (green), MML – middle molecular layer (magenta), OML – outer molecular layer (red). Suprapyramidal and infrapyramidal regions are indicated. In (A) and (B), directional arrows represent 1 mm.

In order to grow dentate gyrus granule cell (GC) dendritic tree structures within their structural context, we first generated a parameterized volume representing the dentate gyrus (DG) shape. Smoothed surfaces for the boundaries of the DG granule cell layer (GCL) and molecular layer (ML) were obtained from a recent high-resolution, 3D serial reconstruction of the rat hippocampus [24]. Parametric 2D manifolds were then fitted to these boundary surfaces (Figure 1A; see also Methods for detailed equations) in order to provide a coordinate system in which depth in the GCL and ML as well as the septo-temporal and infra- versus suprapyramidal axes are mapped. This in turn enabled the subdivision of the ML volume into inner (IML), middle (MML), and outer molecular layers (OML) using intermediate surfaces, since several aspects of GC morphology have previously been associated to these reference structures. The resulting model DG closely matched the structural features of the experimentally reconstructed volume (Figure 1B). The model DG had the same overall GCL volume, 3.78 mm^3^, and ML volume, 9.02 mm^3^, as the experimental reconstruction. Also, the ML width throughout the structure, 247±33 µm, closely matched a previous experimental measurement, 249±33 µm [42]. Slices from the model DG possessed the characteristic curved structure of the biological dentate gyrus, which is known to be more “V”-shaped in the septal region and “U”-shaped in the temporal region (Figure 1C). The volume created by the parametric surfaces thus served as a realistic structural context within which to drive GC dendrite generation. Furthermore, the parametric character of the surfaces subsequently enabled the mathematical tractability of the transformation between a planar two-dimensional sheet and the curved two-dimensional manifolds in 3D space.

### Generation of synthetic dendritic trees

**Figure 2:**
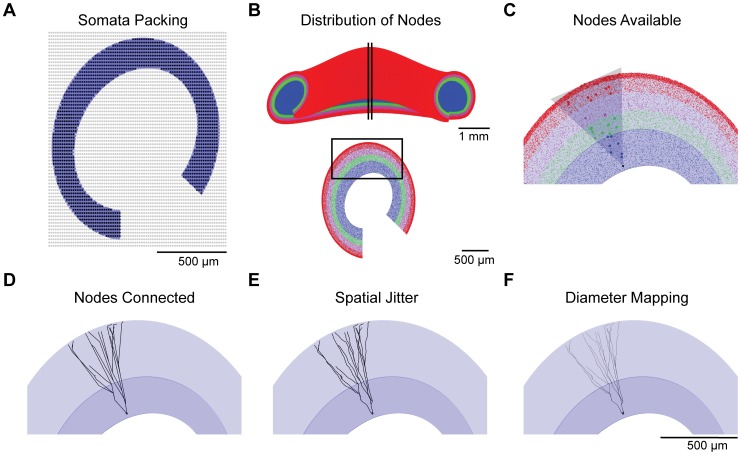
Individual steps in the generation process of granule cell dendritic morphologies. (**A**) Spheres with a 12.54 µm diameter were distributed on a closed hexagonal grid in the volume of the entire GCL, and packed spheres inside the GCL (black dots) were kept and outside the GCL (grey dots) were discarded, leaving a tightly packed arrangement for somata within the GCL volume. A 20 µm transverse slice with one layer of somata is shown for illustration. (**B**) Target points are distributed in the GCL (blue), IML (green), MML (magenta), and OML (red). Model dentate gyrus full structure (top) and a transverse 200 µm slice (bottom) are shown for illustration. The location of the slice is depicted by the vertical black lines. The black box represents the viewpoint for subsequent panels. (**C**) Target points lying within an elliptical cone are selected from the pool of available target points, and a subset of these target points is selected to generate the tree (larger points). (**D**) Target points are connected to minimize total dendritic length and path lengths in the tree. (**E**) Spatial jitter is added to reproduce the tortuosity of branches observed in real reconstructions. (**F**) Diameter values consistent with a quadratic taper toward the dendritic tips were mapped onto the resulting synthetic dendritic morphologies.

The dendritic field spanned by the GC dendritic tree can be approximated by an elliptical cone [41]–[43]. GC trees can be synthesized by connecting a somatic coordinate to target points distributed in such cone-like volumes while minimizing total dendrite length as well as path lengths within the dendrite [41]. To generate the complete GC forest, somata were first distributed within the GCL. The rat DG GCL is estimated to contain approximately 1.2 million tightly-packed GCs [44], [45], and the GC soma is an ellipsoid with an average width of 10.8 µm and height of 18.6 µm [42]. For the purposes of this study, spheres with 12.54 µm diameter corresponding to the volume of an average GC ellipsoid soma were arranged on a large hexagonal grid. Those spheres with any portion located outside of the GCL volume were discarded, and the remaining spheres were selected as somata for the GC population (Figure 2A). In this way, 1.19 million somata separated by a 3.5 µm distance were well-packed within the GCL volume. An elliptical cone could now be placed at each of these soma locations to select the target points necessary to grow GC dendritic trees within the ML boundaries.

The optimal wiring algorithm connects points by performing a dual minimization of total dendritic length and path lengths, under a constraint (balancing factor *bf*) that weighs the importance of one over the other. Low values for *bf* lead to strongly minimizing the total wiring which can result in long conduction paths to the soma, while larger *bf* values lead to trees with short conduction times. Target points for all cells were first distributed in the GCL and ML according to proportions estimated from experimentally reconstructed dendritic trees (Figure 2B, see also Figure S1). Points within each elliptical cone (Figure 2C, shaded area) were then isolated, and a subset of these points (Figure 2C, larger dots) was selected to result in realistic numbers of branch and termination points per layer when connected. These target points were then connected using the optimal wiring algorithm, which results in specific portions of target points becoming branch, continuation, or termination points in the tree depending on the balancing factor [46] (Figure 2D). Spatial jitter of two different spatial frequencies was added to reproduce the tortuosity of real dendritic trees (Figure 2E; see Methods). Finally, a realistic quadratic tapering in diameter was mapped onto the dendritic topologies (Figure 2F), based on both the tapering present in real granule cells and previous work showing that a quadratic taper optimizes synaptic democracy [47], or the equalization of current transfer between all dendritic locations and the root. This process was then repeated for each GC in the DG, varying the parameters to reproduce the variability in the resulting population (details in Methods).

### Validation of synthetic dendritic trees

**Figure 3:**
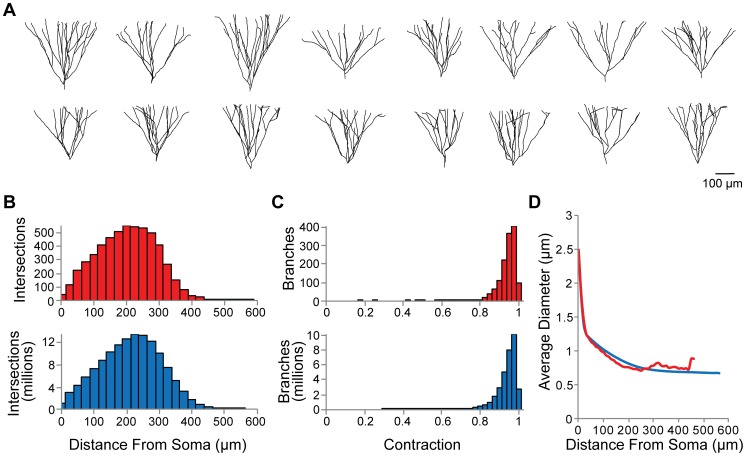
Validation of synthetic dendritic morphologies. (**A**) Example reconstructed (top row) and synthetic dendritic topologies (bottom row). (**B**) Sholl analysis plots for reconstructed (red) and synthetic dendritic trees (blue). (**C**) Contraction value distributions (ratio between Euclidean distance and path distance) for all branches in reconstructed (red) and synthetic dendritic trees (blue). (**D**) Average diameter versus distance from the soma for reconstructed (red) and synthetic dendritic trees (blue).

Synthetic GC dendritic trees were statistically and visually indistinguishable from real GCs. Since the generative wiring algorithm connects target points to form tree structures, it is an important validation of the procedure that both the laminar distribution of branch points and of dendritic length in the synthetic GC population matched the experimental data [42] (Table 1). Example dendritic topologies are shown in Figure 3A. Experimental reconstructions and synthetic dendritic trees had similar branching properties, exemplified by the classical Sholl analysis [48] (Figure 3B), for which the number of intersections between the dendrite and a sphere of increasing diameter centered on the dendrite root are counted. Also, the distributions for contraction values (the ratio of Euclidean distances and path distances for all branches in the tree) were similar between reconstructed and synthetic GCs (Figure 3C), validating the balancing factor between costs of total dendrite length and path distances in the synthetic trees as well as the added spatial jitter. Because diameter measurements are not available for our reference GC morphologies [42], the diameter tapering was constrained to a more recent set of experimental reconstructions [49] independent of the context-dependent study. The match of the diameter tapering between reconstructed and synthetic GCs is visualized in Figure 3D. The branching structure and diameter tapering of the synthetic trees were thus indistinguishable from experimental reconstructions.

**Table 1:** Laminar distribution of branch points and total dendritic length for the synthetic GC population and experimental reconstructions.

	Percent Branch Points		Percent Total Dendritic Length	
Sublayer	Synthetic Population	Experimental Values	Synthetic Population	Experimental Values
IML	61±14	63±14	29±6	30±7
MML	27±13	27±14	32±3	30±7
OML	12±7	10±7	39±5	40±7

Branch points and dendritic length in the GCL were included in the IML values, as was done in the experimental study.

### Matching known context-dependent features

**Figure 4:**
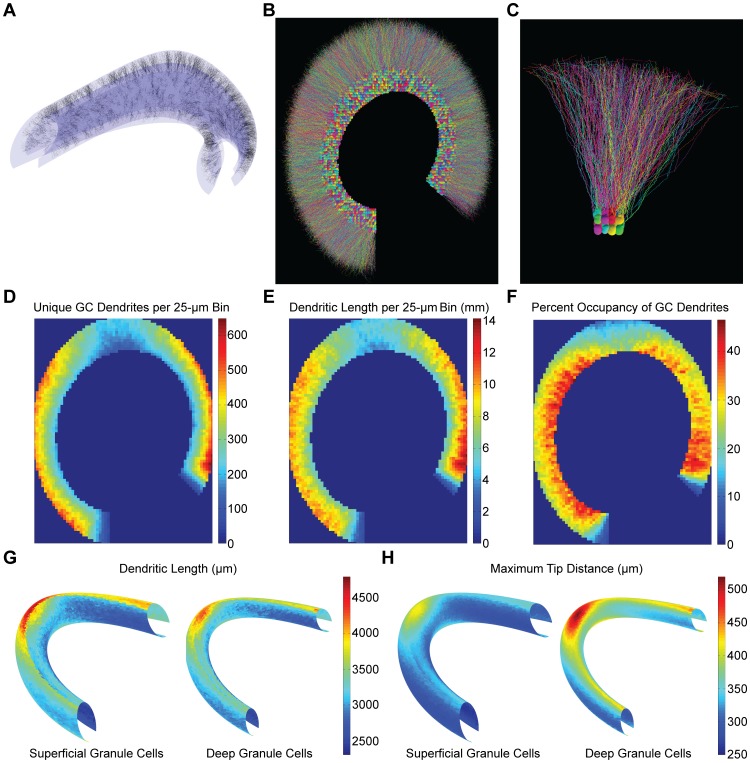
Population analysis of all synthetic granule cells in a rat dentate gyrus. (**A**) Visualization of the boundary surfaces of the dentate gyrus model structure (GCL and ML) and 1,000 synthetic dendritic trees. (**B**) Rendering of the complete morphologies for somata of all granule cells in a 20 µm transverse slice from the center of the model dentate gyrus. (**C**) Rendering of 48 granule cells from the crest of the slice in (B). (**D**) Number of unique granule cells with dendrites reaching into a given cube (25×25×25 µm) of molecular layer volume. The displayed 25-µm slice is located at the center of the model dentate gyrus. (**E**) Density of granule cell dendritic length in the same cubes as (D). (**F**) Percent volumetric occupancy of granule cell dendrites in the same cubes as (D). (**G**) Average dendritic length plotted against the position in the granule cell layer for deep and superficial granule cells. (**H**) Same as (G) but for the average maximum distance between the soma and dendritic tips.

The complete forest of 1.19 million synthetic GC dendritic trees was constructed by varying the parameters in the generation process (i.e., cone radii, number of stems, total number of nodes, laminar distribution of nodes, balancing factor, amplitude of spatial jitter, and diameter taper) for each individual GC. The resulting properties of the complete population matched values from reconstructed granule cells (Table 2), with small differences arising from a different relative composition of GCs from context-dependent subgroups. Significant differences have been described in GC dendritic morphology depending on the location of the soma within the GCL, i.e. for GCs with somata in the suprapyramidal versus infrapyramidal blade as well as in the deep versus superficial parts of the GCL [42]. Accordingly, choosing parameters in the generation process based on the location of each generated granule cell somata allowed for each statistically significant context-dependent difference reported previously to be recreated in the synthetic tree population (Table 3, p<0.001 for all comparisons, Student's t-test). For example, the balancing factor for the suprapyramidal deep granule cells was set to 0.9 to match the higher maximum branch order, which was lower than the 1.35 value used for suprapyramidal superficial granule cells and the 1.22 value used for infrapyramidal granule cells. The lower balancing factor in the suprapyramidal deep subgroup signifies that minimizing dendritic length is more important for these cells. A complete and realistic population of 1.19 million context-dependent GC dendritic trees was created that matched the observed biological variability and recreated context-dependent differences, in addition to fitting within a realistic three-dimensional DG structure (Figure 4A–C). The distribution of these trees within the neuroanatomical space enables the study of the input organization and spatial occupancy of the complete GC forest.

**Table 2:** Overall properties for the synthetic GC population and experimental reconstructions.

Parameter	Synthetic Population (n = 1,185,178)	Experimental Values
# Dendrites	1.8±0.9	1.9±1.4^1^
# Dendritic Branches	28±5	29±7^1^
Max Branch Order	5.7±0.8	5.7±0.7^1^
Transverse Spread (µm)	309±77	325±76^1^
Longitudinal Spread (µm)	173±40	176±42^1^
Total Dendritic Length (µm)	3,357±691	3,221±540^1^
Mean Pathlength to Terminal Tips (µm)	378±62	346±60^2^
Mean Intermediate Branch Length (µm)	86±18	72±17^2^
Mean Terminal Branch Length (µm)	149±32	148±38^2^
Topological Asymmetry	0.45±0.02	0.41±0.02^2^

1 Reported literature value (n = 48).

2 Extracted from experimental reconstructions (n = 43).

**Table 3:** Generation process recreates location-specific differences observed between subgroups of granule cells in experimental reconstructions.

Parameter	Subgroup	Synthetic Population	Experimental Values
# Dendritic Branches	Suprapyramidal	30±5	31±5
	Infrapyramidal	26±4	27±4
Total Dendritic Length (µm)	Suprapyramidal	3,580±671	3,478±482
	Infrapyramidal	3,071±606	2,793±314
# Dendrites	Suprapyramidal Superficial	2.4±1.0	2.4±1.3
	Suprapyramidal Deep	1.5±0.7	1.5±0.7
Max Branch Order	Suprapyramidal Superficial	5.5±0.7	5.5±0.9
	Suprapyramidal Deep	6.4±0.7	6.4±1.0
Transverse Spread (µm)	Suprapyramidal Superficial	374±69	378±70
	Suprapyramidal Deep	290±53	293±53
	Infrapyramidal Superficial	302±57	311±59
	Infrapyramidal Deep	243±60	244±64

### Population-level analyses

In the following, we show how simple analyses that become possible with such a model can be informative about the network constituency in the hippocampus and about the location-specific distances of dendritic structure within the DG volume. An important question regarding the connectivity in the circuit is to know how many unique GCs an axonal arborization would reach within a given volume of the molecular layer. This can now simply be visualized as exemplified for a sample transverse slice from the center of the dentate gyrus (Figure 4D), divided into 25 µm cubic volumes. The overlap of dendrites from unique GCs in each sample volume (312±114 GCs, range 45 to 650) is a small portion of the 1.19 million GC population, signifying that the macroscopic neuroanatomy of the DG promotes a sparse connectivity. This large range also results in a diverse amount of complexity required for an axon to arrive within 5 µm of all GCs in the sample cubic volumes (64±22 branch points, range 17 to 142, see Methods for axon construction details). The distribution was location dependent, as there was a greater overlap in unique GC dendrites in the OML versus the IML, which coincides with the increased dendritic length in the OML (Table 1) and an increased cable density, i.e. dendritic length per volume (Figure 4E). The volume occupied by the GC forest, on the other hand, decreased toward the OML (Figure 4F), signifying that the increased cable density in the OML does not counteract the diameter tapering implemented into synthetic GC dendrites. The overlap of unique GCs, cable density, and volume occupied by GC dendrites were larger in the supra- and infrapyramidal blades as opposed to the crest, which is in accordance with the increased ML volume at the crest reported in the experimental reconstruction [24]. There were significant positive correlations between the overlap of unique GC dendrites, cable density, and volume occupied (Figure S2, p<0.001). However, the correlation between the number of unique GC dendrites and cable density (r = 0.95) was much stronger than the correlation between the number of unique GC dendrites and volume occupied or the cable density and volume occupied (r = 0.27 and 0.50, respectively). This likely resulted from the implemented variability in diameter tapering.

While the occupancy features impact strongly on network connectivity, measures of dendritic morphology that vary along with spatial coordinates in the DG impact strongly on the electrotonic constituency and resulting dendritic computation and synaptic integration in individual GCs. Using our complete population model, we can compare simple measures such as total dendritic length (Figure 4G) and maximum tip distances (Figure 4H) in a location-dependent manner. Even ignoring the difference between GCs from the suprapyramidal and infrapyramidal blades since these were directly incorporated into the model, the total dendritic length varied by a factor of 2× between the most distal septal or temporal tips of the DG as compared to the center of the model. The transverse axis, or size of the “C”-shape, is higher toward the center compared to the septal and temporal tips in the experimental reconstruction [24], so the GCs in the center have an increased length in order to reach the outer edge of the OML. GCs with somata deep in the GCL had less variability in total length compared to the GCs with somata in more superficial parts of the GCL (Figure 4G). As expected, maximum tip distances in deep GCs were longer than in superficial GCs (Figure 4H).

We therefore have provided here simple measures linking the macroscopic scale of the DG volume with the microscopic details of single neuron morphologies and extracted useful information for network connectivity and neural computation. In future studies, novel population-level measures can be designed and tested utilizing this framework as a foundation.

## Discussion

In the present study, we used a realistic structural context based on a reconstructed rat dentate gyrus [24] to drive the generation of dendritic trees with a recently developed algorithm based on optimal wiring constraints [47], [50]. By varying the relatively few parameters in the generation process, we were able to reproduce the observed biological variability in the morphology of dentate gyrus granule cells and match key location-specific differences. While some properties were obtained from parameter optimization, several features were emergent and not the result of direct parameter constraints, including the total dendritic length, branch lengths, path lengths, and asymmetry in Table 2 as well as the Sholl intersections in Figure 3. In addition, all population-level measures, such as the cable density, are emergent properties.

The set of synthetic dendritic trees represents the largest collection of realistic morphologies to date, a complete forest of 1.19 million granule cell dendritic trees, with each tree requiring less than two seconds to be constructed. The method that we devised enables population-level analysis, and we can link the larger neuroanatomical features with the resulting branching characteristics. Due to the small number of existing reconstructions that are registered to a macroscopic context and the limited information about the properties of granule cells in the crest, granule cells were split into subgroups differentiating infrapyramidal versus suprapyramidal and deep versus superficial granule cells based on previous reconstructions [42], and a single balancing factor parameter was specified for each group. As more context-aware reconstructions become available, this abrupt transition can be modified to create a more continuous variation of the parameters. The speed conferred by utilizing parallel computing in the generation process and the relatively few parameters involved provide flexibility to incorporate future experimental observations to improve the model.

While the current model provides a valuable framework for the exploration of macroscopic and microscopic neuroanatomical links, there are inherent simplifications that deviate from the biological condition that should be improved upon in future studies. The current generation process allows for multiple dendrites to occupy the same point in space, so a form of avoidance could be implemented into the spatial tortuosity, instead of solely low-pass filtered noise, in order to create a more realistic spatial occupancy. In addition, the packing of spherical somata can be improved to implement the variable and tightly-packed elliptical somata observed in experimental studies [42]. The current study also does not include the newborn granule cells, which constitute approximately 10% of the total granule cell population [51], transiently exhibit basal dendrites [52]–[54], and possess a significantly smaller total dendritic length [53]. This subpopulation has recently come under intense focus for their unique participation in hippocampal network functions [55]–[57], and the neuroanatomical properties of this subpopulation could be contrasted with the more numerous mature granule cells constructed in this study.

The linking of macroscopic and microscopic neuroanatomy presented in this study provides a framework that can be expanded upon with additional cell types and axons, but it also provides an avenue to link neuroanatomical features with electrophysiological function. The breadth of anatomical data being collected, including recent experimental reconstructions of excitatory mossy cells [49] and inhibitory interneurons [58], [59] in the dentate gyrus, will make it possible to construct even more biologically realistic DG models. In addition, the context-driven generation methodology can be applied to axons to create realistic connectivity for comparison to the growing connectomics literature. As noted in the introduction, dendritic morphology can have a dramatic impact on electrophysiological function, and the framework provided in this study allows for this relationship to be studied on the level of the complete population. All generated morphologies can be exported to simulation environments [60] for the insertion of ion channel conductances or other biophysical mechanisms. For the example case of granule cells, the measured properties of dendritic integration [61], [62] and action potential initiation [63] should serve as valuable constraints. This structure and function relationship can eventually be linked to both the macroscopic neuroanatomical and network context.

## Methods

The model dentate gyrus structure and granule cell synthetic trees were created and analyzed in MATLAB using the TREES toolbox [41], [60] on University of California Irvine's High Performance Computing cluster. The model structure and generation process will be made available at ModelDB (http://senselab.med.yale.edu/ModelDB/). The standard deviations for the literature values [42] were determined by multiplying the reported standard error by the sample size. All values are presented as mean ± standard deviation.

### Model dentate gyrus structure

The following parametric equations defined the layer boundaries:
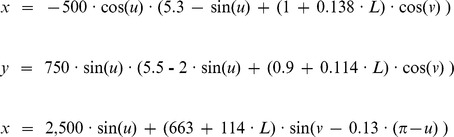
where *v* defined the “C”-shape and ranged from −0.23π to 1.425π, *u* defined the septotemporal extent and ranged from 0.01π to 0.98π for the GCL and −0.016π to 1.01π for the ML, and *L* defined the layer and was −1.95 for inner GCL, 0 for outer GCL, 1 for IML, 2 for MML, and 3 for OML. The experimental reconstruction GCL volume was calculated based on the average 0.6 GCL to hilus volumetric ratio and their combined volume of 6.30 mm^3^ [24]. The experimental value for the molecular layer width was determined by combining the means and standard deviations for the infrapyramidal and suprapyramidal group measurements (240±17 and 254±3, respectively) reported in a previous study [42]. The model ML width was determined by distributing 2 million points on the outer GCL and OML boundary, and then calculating the closest distance to the OML boundary from 10,000 randomly sampled outer GCL points.

### Synthetic dendritic tree generation

In order to recreate the context-dependent differences in the synthetic tree population, the size of the elliptical cone, number of stems, total number of nodes, and balancing factor governing the wiring were modified based on the location of each soma. Superficial neurons were defined as having somata in the half of the GCL closest to the ML, whereas deep neurons had somata in the half farthest from the ML. The infrapyramidal/suprapyramidal split was located halfway around the characteristic “C”-shape of the transverse slice of the dentate gyrus, which was defined by the midpoint of the *v* parameter in the GCL boundary equation. The number of stems was set by sampling from a truncated Poisson distribution and ranged from 1 to 4, as observed in experimental reconstructions [42], [49]. The elliptical cone was oriented by pointing the center axis toward the closest of two million points distributed on the OML boundary and orienting the longitudinal and transverse elliptical cone radii within the structure. The transverse spread of generated GCs was analyzed by orienting cells based on their mean transverse axis and measuring the distance between the outermost dendritic tips. The widest spread in the majority of granule cells is reported to be close to the transverse axis [42], so the elliptical cone transverse radius was set greater than the longitudinal radius in the generation process. All trees were resampled to a 5 µm fixed segment interval, and low-pass filtered homogenous spatial noise was applied to all points similar to previous methods [41], using length constants of 10 µm and 50 µm. Diameter mapping was implemented using a variable quadratic tapering from previous studies [41], [47] and adding an additional scaling function exp(*x*) – 1, where *x* is the distance from the soma, to better approximate the initial diameter taper close to the soma. Experimental reconstructions [42] used in the target point laminar distribution estimation (see Supporting Information) and synthetic tree validation were obtained from the www.NeuroMorpho.Org database [7].

### Population-level analyses

The ray-tracing images in Figure 4B–C were created with the Persistence of Vision Ray tracer (POV-ray) software (http://www.povray.org/download/). The location of dendrites within each 25 µm cube was determined by testing the points in each dendritic tree, which specify the center of each segment. Because the granule cell dendritic trees were resampled at 5 µm before the spatial jitter addition, the length and volume measurements within each cubic volume are approximations. To get an estimate of the complexity required for an axon to contact all granule cell dendritic trees invading each cubic volume, random points were selected for each cubic volume and connected using the optimal wiring algorithm with a balancing factor of zero (to minimize total dendritic length). The number of target points was increased until the simulated axon reached within 5 µm of all granule cell dendritic trees present in the each volume. The results from 10 different random collections of target points were averaged together to determine the complexity required (number of branch points) for each cubic volume. In order to map the dendritic length and maximum tip distance onto the GCL, triangulations of the inner and outer GCL surfaces were created with 5000 faces, and the closest face for all trees was determined. The values for the trees associated with each respective face were then averaged together.

## Supporting Information

Figure S1 Branch and termination point distributions estimated from experimental dendritic tree reconstructions. (**A**) Overlay of rotated three-dimensional reconstructions of 43 granule cell dendritic morphologies. (**B**) Size-normalized dendritic morphologies scaled to the average limits in all three dimensions. (**C**) Distribution of branch and termination points in each layer. GCL – granule cell layer (blue), IML – inner molecular layer (green), MML – middle molecular layer (magenta), OML – outer molecular layer (red).
(TIF)

Figure S2 Correlations between occupancy measures. (**A**) Number of unique granule cells with dendrites reaching into a given cube (25×25×25 µm) versus the cable density. Cubic volumes are the same as in Figure 4D–F. (**B**) Number of unique granule cells with dendrites reaching into a given cube versus percent volumetric occupancy in the same cubes as (A). (**C**) Cable density versus percent volumetric occupancy in the same cubes as (A).
(TIF)

Text S1 Estimation of target point laminar distribution.
(DOC)
