# Supplementary figures and images for: Linking Macroscopic with Microscopic Neuroanatomy Using Synthetic Neuronal Populations
Source: PLoS Comput Biol. 2014 Oct 23;10(10):e1003921. doi: 10.1371/journal.pcbi.1003921 (PMC4207466; doi:10.1371/journal.pcbi.1003921)

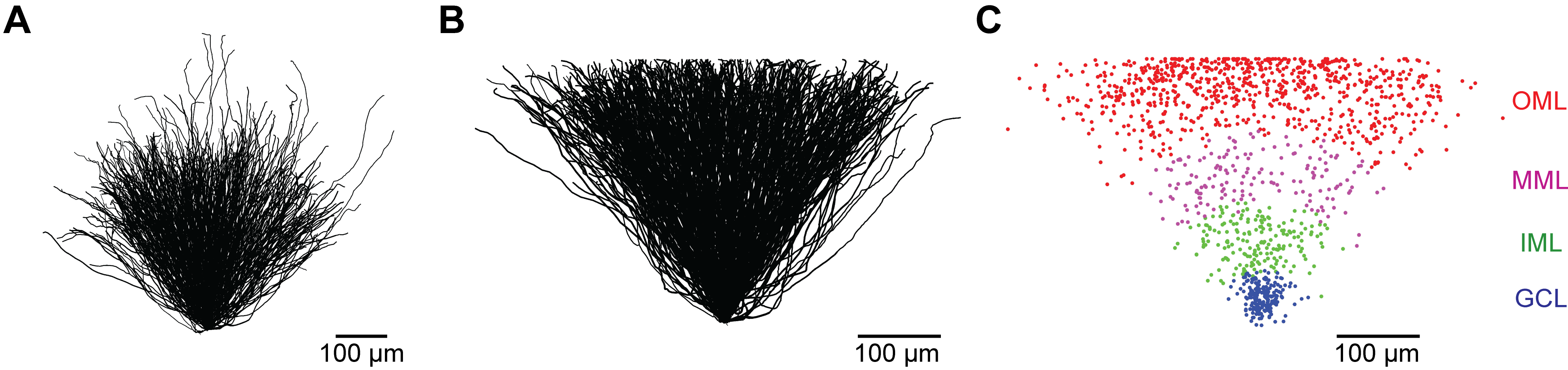

Supplement: Figure S1 — Branch and termination point distributions estimated from experimental dendritic tree reconstructions. (A) Overlay of rotated three-dimensional reconstructions of 43 granule cell dendritic morphologies. (B) Size-normalized dendritic morphologies scaled to the average limits in all three dimensions. (C) Distribution of branch and termination points in each layer. GCL – granule cell layer (blue), IML – inner molecular layer (green), MML – middle molecular layer (magenta), OML – outer molecular layer (red). (TIF) [file pcbi.1003921.s001.tif]

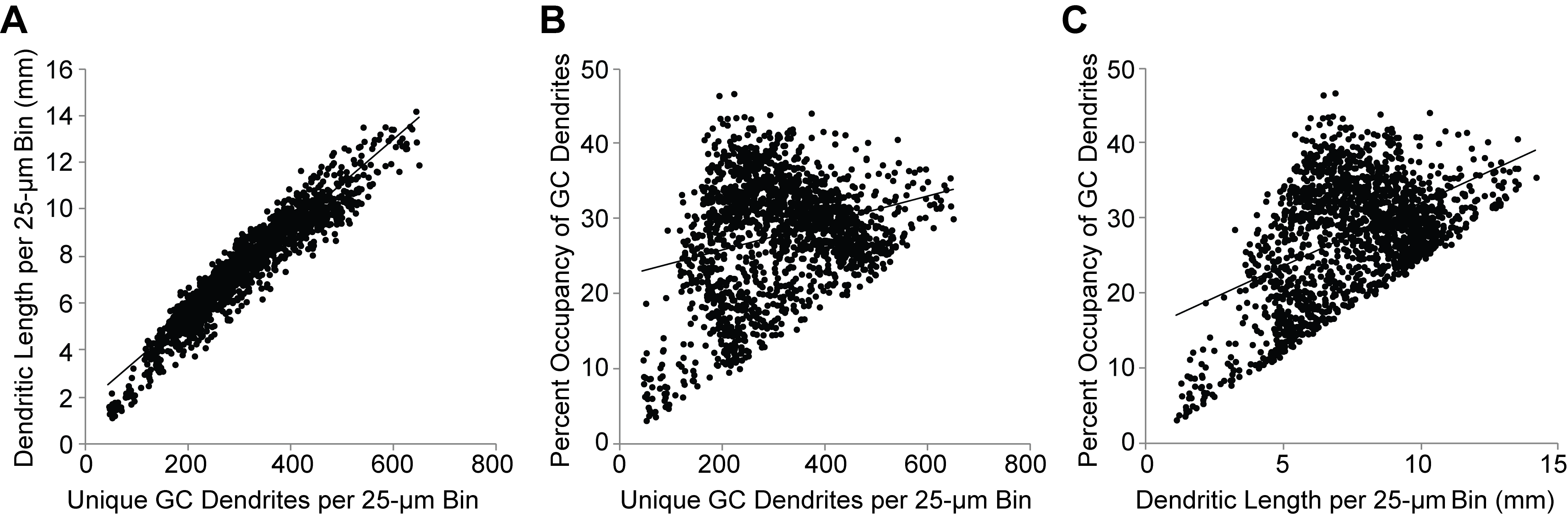

Supplement: Figure S2 — Correlations between occupancy measures. (A) Number of unique granule cells with dendrites reaching into a given cube (25×25×25 µm) versus the cable density. Cubic volumes are the same as in Figure 4D–F. (B) Number of unique granule cells with dendrites reaching into a given cube versus percent volumetric occupancy in the same cubes as (A). (C) Cable density versus percent volumetric occupancy in the same cubes as (A). (TIF) [file pcbi.1003921.s002.tif]
