# Supplementary material for: Linking Macroscopic with Microscopic Neuroanatomy Using Synthetic Neuronal Populations
Source: PLoS Comput Biol. 2014 Oct 23;10(10):e1003921. doi: 10.1371/journal.pcbi.1003921 (PMC4207466; doi:10.1371/journal.pcbi.1003921)
Supplement: Text S1 — Estimation of target point laminar distribution. (DOC) [file pcbi.1003921.s003.doc]

**Text S1**

**Laminar distribution of target points for synthetic granule cell generation**

The optimal wiring algorithm connects input target points and allocates each point as a branch, continuation, or termination point in the final structure. The number of target points and their laminar distribution were thus required to generate dendritic morphologies in the model dentate gyrus structure. These parameters were estimated from three-dimensional dendritic reconstructions of 43 granule cells, which were performed in 400-µm slices and contained no cut dendrites in the IML and two or less cut dendrites in the MML and OML . Only branch and termination points were summed together to estimate target points in the reconstructions, because continuation points are a part of the generation process and do not have significance in the reconstructions. The dendritic reconstructions had an average of 32 total branch and termination points, which slightly underestimates the average number of target points required in the generation process due to the lack of continuation points. To extract the laminar distribution estimate, reconstructions were rotated and size-normalized. All experimental reconstructions were originally performed perpendicular to the longitudinal axis of the dentate gyrus, so they were only rotated in the transverse plane to orient their mean transverse axis and then their outermost dendritic tips (Figure S1A). The reconstructions were then scaled to the average limits in all three directions to produce size-normalized dendritic trees (Figure S1B), as done previously . Branch and termination points were then grouped into layers based on their distance from the base of the normalized trees, choosing the maximum normalized tree height as the edge of the molecular layer and the molecular layer width as the average experimentally determined value of 249 µm (Figure S1C). This resulted in estimated proportions of 14%, 14%, 12%, and 60% for the GCL, IML, MML, and OML, respectively. Because virtually all granule cell dendrites extend toward the OML boundary , points in the OML were preferentially distributed in the outer fourth of the layer, according to a 2:1 ratio measured in the estimation process. This weighted distribution in the outer portion of the OML was crucial to achieve the correct laminar distribution of the total dendritic length (see Table 1). The inclusion of the billions of points available in the model dentate gyrus structure as possible target points in the generation process would be computationally expensive, even with parallel computing, and is not necessary for the 1.19 million neurons being generated. The complete set of points located within the model GCL and ML was first determined at a 1 µm interval, and a subset of 200 million random points was chosen from the GCL and the sublayers of the ML according to the laminar distribution extracted above. The selection of target points in the final generation process was 14 ± 4.1% in the GCL, 13 ± 4.1% in the IML, 11.5% in the inner OML, 48% in the outer OML, and the remaining percentage, on average 13.5%, in the MML.
